# Pathophysiology and Therapy of Associated Features of Migraine

**DOI:** 10.3390/cells11172767

**Published:** 2022-09-05

**Authors:** Maria Dolores Villar-Martinez, Peter J. Goadsby

**Affiliations:** 1 Headache Group, Wolfson CARD, Institute of Psychiatry, Psychology and Neuroscience, King’s College London, London WC2R 2LS, UK; 2 NIHR King’s Clinical Research Facility, SLaM Biomedical Research Centre, King’s College Hospital, London SE5 9RS, UK; 3 Department of Neurology, University of California, Los Angeles, CA 90095, USA

**Keywords:** migraine pathophysiology, nausea, osmophobia, phonophobia, vertigo, allodynia

## Abstract

Migraine is a complex and debilitating disorder that is broadly recognised by its characteristic headache. However, given the wide array of clinical presentations in migraineurs, the headache might not represent the main troublesome symptom and it can even go unnoticed. Understanding migraines exclusively as a pain process is simplistic and certainly hinders management. We describe the mechanisms behind some of the most disabling associated symptoms of migraine, including the relationship between the central and peripheral processes that take part in nausea, osmophobia, phonophobia, vertigo and allodynia. The rationale for the efficacy of the current therapeutic arsenal is also depicted in this article. The associated symptoms to migraine, apart from the painful component, are frequent, under-recognised and can be more deleterious than the headache itself. The clinical anamnesis of a headache patient should enquire about the associated symptoms, and treatment should be considered and individualised. Acknowledging the associated symptoms as a fundamental part of migraine has permitted a deeper and more coherent comprehension of the pathophysiology of migraine.

## 1. Introduction

Migraine has been traditionally associated with the core symptom, headache [1]. Photophobia and vomiting, two of the canonical symptoms associated with migraine [2], are also widely accepted features of the typical migraine attack, as understood classically by patients and physicians [3]. However, reducing the understanding of migraine to a few symptoms would be as simplistic, perhaps, as reducing Parkinson’s disease to tremors.

The way that migraineurs deal with their attacks provides valuable information about hypersensitivity to sensorial stimulation, including avoiding movement, light, sounds, touch or smells [4]. These are usually subjective, unpleasant experiences, unshared by family, friends or colleagues. Consequently, migraine patients presenting associated symptoms as prominent features can usually be labelled as sensitive. The Greek translation for sensitive, Ευαίσθητος “evahistos”, can be separated into the following two parts: the prefix meaning good or well, and the rest meaning sense or perception. However, any positive connotation of the term has nowadays dissipated. Many of these “evahistic” manifestations can actually be the main symptom of the clinical picture in a patient with migraine, and imply a higher disability [5]. Migraine patients with sensory hypersensitivity may have more attention difficulties during daily activities [6], or more cranial autonomic symptoms associated to the headache [7], and the response to preventive treatments may vary [8]. Exogenous factors, such as stress, obesity, intestinal microbiota and even parental behaviour, have been speculated to play a role in the chronification and sensitization process [9,10,11,12].

In recent years, the study of non-headache symptoms has been useful in demonstrating their important role, yet independence from pain, in the pathophysiology of migraine. In this paper, we will focus on some frequently disabling associated symptoms, such as nausea, osmophobia, phonophobia, neuro-otological manifestations and cutaneous allodynia, and will spare comments on some premonitory-like symptoms, such as yawning. Photophobia has recently been reviewed elsewhere [13], and the usually omnipresent symptom in migraineurs, movement sensitivity, could be explained by some mechanisms that are commented on below.

## 2. Nausea and Vomiting

### 2.1. Nausea in Migraine and Conditions Related to Migraine

Nausea is one of the symptoms associated with migraine that is considered canonical, according to the International Classification of Headache Disorders, 3rd Edition (ICHD-3) [2]. Ictal and interictal nausea has a high impact on quality of life and economic cost [14,15], and is the second most bothersome migraine symptom, reported in 28% of patients, exceeded only by photophobia [16].

Up to half of the people with episodic migraine suffer from nausea in more than half of their headache episodes, and the attacks were accompanied by more headache symptoms and a higher impact, compared to patients with less frequency of nausea. The majority of those reporting high-frequency nausea were women [17] and had an increased risk of developing chronic migraine in 2 years [18].

Having migrainous biology could result in patients having more disability when presenting with other disorders that are generally associated with nausea and vomiting.

#### 2.1.1. Cyclic Vomiting Syndrome

It is well known that there is a strong link between migraine and cyclic vomiting syndrome [19], with similar associated symptoms during the attacks, and triggers, as reported by the patients [20,21]. Both nausea and cyclic vomiting syndrome patients have a decreased connectivity between the sensorimotor network and the insula, which manages viscero-sensory processing [22] and may be regulated by the endocannabinoid system [23]. Cannabis can act as a pro-emetic or antiemetic and can cause cannabis hyperemesis syndrome, which shares similar features to cyclic vomiting syndrome [24], and whose recommended treatment is cannabis cessation [25]. Remarkably, the management of cyclic vomiting syndrome consists predominantly of treatments also used for migraine [26].

#### 2.1.2. Motion Sickness

Motion sickness and migraine may share a similar pathophysiology, as patients with motion sickness have a robust migrainous biology [27] and around half of migraineurs present with motion sickness, in comparison with 20% of those with non-migrainous headaches [28]. Patients with “migrainous vertigo” had an improvement in severe motion sickness following rizatriptan [29]. Nociceptive stimulation in the trigeminal area is capable of increasing nausea during motion sickness caused by optokinetic stimulation [30], whereas nausea did not increase following extra-trigeminal nociceptive inputs [31]. Having a history of migraine has also been associated with developing post-operative nausea [32,33], and having motion sickness and being a female are independent risk factors for post-operative vomiting [32,34].

#### 2.1.3. Pregnancy

Pregnancy is potentially a particularly disabling period for women with migraine. During pregnancy, one third of migrainous women require hospitalization due to hyperemesis gravidarum, and almost forty percent of women with hyperemesis reported migraine headaches [35].

Finally, a migrainous background may determine the quality of life related to nausea in palliative care, and migraine preventive treatments serve as efficacious relief in treating incoercible nausea in terminal patients with a history of migraine [36].

### 2.2. Neuroanatomy and Neuropharmacology

There is a matrix of neuro-anatomical structures involved in the onset and control of nausea, as well as several neurotransmitters that have been the main targets of antiemetic and acute treatment schemes.

Dopamine has been the main compound implicated in the pathophysiology of nausea associated with migraine, at least since the 1970s [37]. Patients with migraine are sensitive to dopaminergic pharmacological agents [38,39,40] and develop nausea and other classically considered dopaminergic symptoms, such as yawning, not necessarily accompanied by headache [38,40]. This propensity may entail a genetic predisposition, and a particular allelic distribution was found to be significantly different for the D_2_ dopamine receptor in a subpopulation of migraineurs with prominent dopaminergic symptoms [41]. Among the dopaminergic symptoms, nausea, unlike yawning, is considered post-synaptic, and is triggered by apomorphine and inhibited by domperidone, which targets D_2_ receptors [40]. Dopamine may also regulate headache pain, as dopaminergic neurons play a role in nociceptive control by modulating trigemino-vascular neurons [42].

Serotonin also has a major role in nausea, with the receptor 5-hydroxytryptamine- 5-HT_3_ as the main target not only of modern antiemetic pharmacological compounds, but also of natural antiemetics used for centuries, such as the gingerol compounds contained in ginger [43].

Hyporexia during headaches may be explained by the loss of appetite that can be observed during noxious dural stimulation, which activates the nucleus parabrachial and the ventromedial of the hypothalamus, and may be mediated by cholecystokinin [44]. However, nausea can also appear before the headache, during the premonitory phase, in almost a quarter of spontaneous attacks [45]. This percentage was doubled when headache attacks were triggered in a controlled environment [46].

Another intriguing component in migrainous nausea is substance P. Neurokinin 1 (NK-1) receptor antagonists can inhibit vomit produced by central or peripheral stimuli [47], and its central action may be mediated by inhibiting the substance P emetic effect [48], which may take place predominantly in the locus coeruleus [49].

Early pre-clinical experiments are good examples of the extent of anatomical structures that could be involved in the process of vomiting. Monkeys presented vomiting following the electrical stimulation of the olfactory tubercle, amygdala, septum, fornix and the thalamic ventral anterior nucleus [50]. In cats, lesions in the medulla abolished the characteristic pattern of respiratory motor nerve discharge, observed in vomiting [51], induced by emetic drugs and electrical vagal stimulation of abdominal afferents. This study suggested that the regions that control vomiting were localised between the obex and the retrofacial nucleus [52], both localized in the medulla.

In human neuroimaging studies, some brainstem areas showed significant activation with a H_2_^15^O positron emission tomography (PET) scan in the premonitory phase of migraine participants with nausea, including the periaqueductal grey, dorsal motor nucleus of the vagus, nucleus ambiguous and nucleus tractus solitarius [53], as shown in the following paragraphs. Following a rostral-caudal approach, among them, the mesencephalic periaqueductal grey (PAG) deserves a special mention [53].

**Figure 1:**
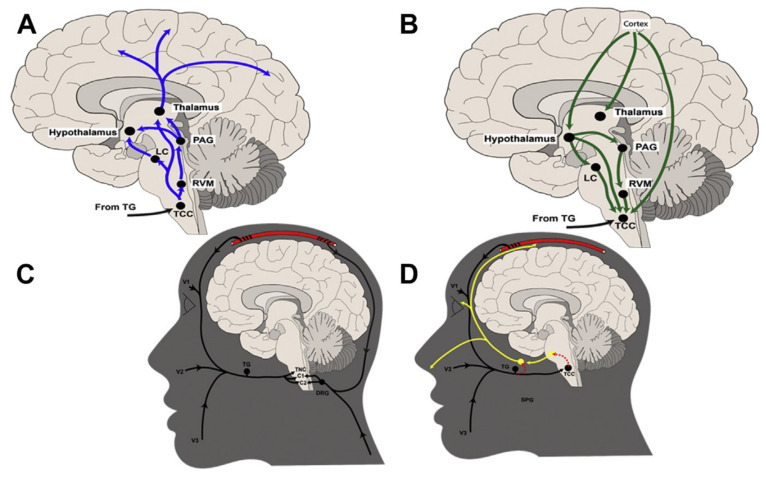
Schematic representation of ascending and descending mechanisms involved in the pathophysiology of migraine, interaction between peripheral and central nervous systems and the trigeminal autonomic reflex. (**A**) Ascending mechanisms; (**B**) Descending mechanisms; (**C**) Connection of dural, cervical and trigeminal inputs in the trigeminocervical complex; (**D**) Potential interfaces between trigeminal and parasympathetic arms of the trigeminal autonomic reflex. Cervical dermatomes (C1, C2); dorsal root ganglia (DRG); locus coeruleus (LC); periaqueductal gray (PAG); sphenopalatine ganglion (SPG); trigeminal ganglion (TG); trigeminocervical complex (TCC) rostral ventromedial medulla (RVM); ophthalmic, maxillary, and mandibular dermatomes of the trigeminal nerve (V1, V2, V3, respectively). Reproduced from Goadsby and Holland 2019 with permission.

PAG has an important role in the descending modulation of the trigeminovascular processes (Figure 1) [54]. PAG has been related to other autonomic sympathetic activity [55,56], emotional perception of pain and aversive behaviours [57,58] cough [59] and breathing control [60]. It is involved in modulating the descending pain pathways [61,62,63]. This modulation has recently been shown to be activated by mu opioids by means of presynaptic disinhibition and reducing GABAergic postsynaptic currents [64]. It is yet unknown whether this area is related to the chronification observed in migraineurs with frequent use of opioids, as commented on below.

More caudal areas in the rostral dorsal medulla were involved, including the dorsal motor nucleus of the vagus [53], which may relax the lower esophageal sphincter [65].

The nucleus tractus solitarius has connections with hypothalamic areas that play a role in autonomic control [66]. Both the nucleus tractus solitarius and dorsal motor nucleus of the vagus conform, along with the area postrema, the dorsal vagal complex, which is one of the main termination sites of the afferent fibres of the vagal nerve [67] and has a high distribution of dopamine D_2–4_ receptors [68]. The area postrema is one of the sensory circumventricular organs with a possible chemoreceptive function, situated outside the blood–brain barrier and connected to the hypothalamus, which is thought to be essential in controlling neuroendocrine functions [69], is rich in type D_2_ dopamine receptors [70] and is the brain area with the higher estimates of substance P [71].

### 2.3. Treatment of Nausea

The treatment of nausea during migraine attacks must be considered in every patient presenting with that symptom. When nausea does not respond to analgesic treatment, specific antiemetic treatment should focus on the pathways of the neurotransmitters described above (dopamine, serotonin, substance P) as main targets for treatment. Nevertheless, acute treatment can be essential in the management of nausea associated with migraine. NSAIDs could be effective in alleviating nausea in patients who have not taken any triptans [72] and there is a recent meta-analysis that supports gepants as an effective treatment for nausea in patients with episodic migraine [73]. Special attention must be paid to patients consuming opioids. Nausea is a recognised side effect following opioid use [74]. Patients with episodic migraines who are exposed to opioids have a twofold risk of migraine chronification [75], a likely reduction in the efficacy of triptans for acute treatment [76] and the issue of developing gastro-intestinal adverse events after long-term consumption [77]. For the treatment of nausea, we have focused on the three main neurotransmitters involved, serotonin, substance P and dopamine.

#### 2.3.1. Serotonin

Triptans are serotonin 5-HT_1B/1D_ receptor agonists, and can help in alleviating nausea, as exemplified by rizatriptan [78,79]. However, having a sensation of nausea pre-treatment predicts a low efficacy response [80], perhaps due to the delay in treatment intake, as discussed in the allodynia section. Ondansetron is a highly-specific 5-HT_3_ receptor antagonist, although there are no randomized-controlled trials on migraine. Granisetron, however, was significantly more effective than placebo for nausea at 30 min [81], and was more effective than metoclopramide as an adjuvant treatment for acute migraine [82].

Ginger could be a reasonable “over the counter” serotonergic therapeutic strategy for patients trying to avoid chemical treatments. It might be effective in lowering nausea according to a meta-analysis of three studies [83], and headache relief similar to that of sumatriptan has been reported in a double-blind, randomized controlled study [84].

#### 2.3.2. Dopamine

Among the several antiemetics available, metoclopramide is an antagonist of dopamine D_2_ receptors and has also an antagonist effect on serotonin 5-HT_3_ receptors [85]. Metoclopramide presents the highest passage of the blood–brain barrier, compared to domperidone or chlorpromazine [86]. Metoclopramide helps with the impairment of gastric motility during migraine attacks, improving the absorption rate of NSAIDs [87], and may also exert its effect as a pain relief agent [88], probably due to its action in the trigemino–cervical complex [89]. However, recent literature found conflicting results as a single therapeutic approach, with either an efficacy similar to that of NSAIDs [90], or no difference of intravenous metoclopramide compared to saline [91]. Prochlorperazine is a phenothiazine antipsychotic with antagonizing effect of dopamine D_2_ receptors, similar to chlorpromazine [87,92] and might be the most effective intravenous antiemetic, which also has a higher risk of extrapyramidal adverse events [93]. Chlorpromazine is also an effective option to consider for the treatment of nausea in emergency settings [94].

#### 2.3.3. Substance P

By inhibiting the substance P pathway, NK-1 receptor antagonists, such as aprepitant, have been used in the treatment of nausea generated by intravenous dihydroergotamine in patients with migraine [95]. NK1 receptor antagonists are potent antiemetics that have been approved for the treatment of severe nausea associated with chemotherapy [96], and are also recommended for cyclic vomiting syndrome, along with ondansetron or triptans [26].

## 3. Osmophobia

The perception of odour is certainly an extremely subjective experience, or we would all be wearing the same perfume. Being perhaps the less studied of the senses, the mechanisms behind the way a fragrance is perceived is not yet fully understood. A brief mention here is appropriate for two interesting theories that were proposed in the twentieth century, involving a lock-and-key system and vibrational wavelengths [97], which have not yet been fully developed.

There are several substances whose consumption or inhalation has been popularly related to headaches [98,99,100,101]. Remarkably, *Umbellularia californica* is a type of tree, commonly known as “the headache tree” [102], which contains umbellulone, a ketone that was reported of being capable of triggering cluster headache-like attacks in a gardener with a history of cluster headaches [103]. It was later discovered that this mechanism was mediated by the activation of the transient receptor potential (TRP) ankyrin 1 (TRPA1) [104,105], followed by the release of calcitonin gene-related peptide (CGRP) [104]. CGRP is also released through the activation of vanilloid receptors, following stimulation with nitric oxide [106] or ethanol [107,108], one of the most relevant cluster headache triggers. TRPA1 has also been involved in the responses to some inhaled chemicals, including the smoke of cigarettes [109], chloride [110,111] hydrogen peroxide-containing substances [111] or formalin, the noxious compound largely used in pain models [112].

It has been reported that up to 70% of migraineurs can develop a headache after the stimulation with some odorants, which happened around 25 minutes following the exposure [113], and there is a case report of migraine improvement following the imposition of mandatory masks in the workplace during the COVID-19 pandemic [114]. Increased sensitivity to smells can be part of the premonitory-like symptoms experienced by migraineurs; therefore, certain smells may be misinterpreted as the trigger for a migraine attack, which might not be a necessary factor for its occurrence [115,116]. As a consequence, the results of studies that assess migraine triggers have debatable interpretations.

Nevertheless, the presence of osmophobia may be related to more florid migraine phenotypes and greater disability, and a scale has been developed recently for the quantification of quality of life related to osmophobia [117]. Migraineurs that present with ictal osmophobia may have more painful headaches [118,119]. Ictal and interictal osmophobia have been associated with a longer history of migraines or high frequency of the attacks, as well as other associated symptoms, such as cranial allodynia [120,121,122], suggesting a central sensitization process [123]. Vomiting can also be more common in the presence of osmophobia [119,121]. Osmophobic migraineurs may also have a higher prevalence of psychiatric comorbidities than those without it [118,124,125,126].

Osmophobia has been proposed as a specific marker, helpful for the diagnosis of migraine [119,124,127,128,129,130,131,132]; however, it is not very sensitive [122]. Around half of the patients with migraines reported an increased sense of smell or reduced tolerability to smells [129,133]. Remarkable examples of patients reporting hyperosmia include the smell of a rose from more than 5 meters of distance, or soap from a different room, and the main scents triggers for osmophobia arose from food, specifically fried food and onions, cigarettes or self-care products, and perfume or paint specifically were reported as triggers [133]. More recently, forty percent of patients with chronic migraine reported osmophobia [134], and a similar number suggested odours or perfumes as potential triggers of a migraine attack [101].

Paradoxically, despite their hypersensitivity to smells, migraineurs have a lower capability for the threshold, identification and discrimination of smells [135,136]. Patients with episodic migraine were found to have a similar olfactory acuity to controls, and furthermore, around one fifth of them developed hyposmia during the attack [137]. Taste abnormalities in migraineurs [133] are a matter of debate [138].

Patients with migraine and osmophobia have neuroanatomical alterations. A significantly reduced volume of the olfactory bulb was observed in 1.5 Tesla MRI, compared to patients with other types of headache [139], and might be more pronounced on the left, in comparison with controls [140]. In migraineurs with reported hypersensitivity to odours, regional blood flow in a study using H_2_^15^O-positron emission tomography was found to be increased in areas of the left piriform cortex and antero-superior temporal gyrus, as compared to controls, both with and without multiple odour stimuli [141]. During odour stimulation, blood flow was found to be decreased in bilateral fronto-temporo-parietal regions, as well as the posterior cingulate gyrus and right locus coeruleus [141]. Another study using fMRI to compare responses to the smell of roses found higher blood oxygen level-dependent activity in the amygdala and insular cortices of the amygdala and also in the midbrain, particularly the rostral pons. However, the smell of roses did not show significant interictal differences compared to the controls [142]. Activation of the amygdala and orbitofrontal cortex might be related, respectively, with the intensity and valence of the smell emotional experience [143]. The amygdala and cingulate cortex also showed abnormal activation in patients with multiple chemical sensitivity [144,145], which is associated with a high prevalence of headache [146] and was observed in up to 20% of migraineurs [147].

Olfactory hallucinations or phantosmia is a hallmark of temporal lobe epilepsy, and currently a no man’s land when it presents in the form of aura. It is a rare symptom, with a reported prevalence of 0.66% in a headache center [148]. The majority of reported cases had normal electroencephalograms that were, however, taken during the interictal period, and usually respond to antiepileptic drugs.

The reported cases showed that the episodes have an average duration of less than 10 min and the onset occurs prior to the migraine attack [148,149]. Patients with symptoms of phantosmia scanned with FLASH and eco-planar imaging MRI techniques showed increased activation of different brain areas associated with the process of the sense of smell, such as the prefrontal, cingulate, temporal or insular cortex MRI activation was inhibited by typical antipsychotics that perform its activity through a wide range of binding receptors [150]. Peripheral blocking activities can alleviate phantosmia [151].

## 4. Neuro-Otological Manifestations

In 1984, Kayan and Hood described how vestibulocochlear symptoms were frequently reported, in up to 60% of patients with migraine, and these can be important or disabling enough for the patient to be the primary reason for referral to a specialist. The incidence of neuro-otological symptoms for migraineurs seemed homogeneous throughout all ages in males, but had a peculiar distribution in females. For women who reported audiovestibular symptoms only when asked during the study, a positive skew distribution could be observed, with the peak situated in the 3rd decade. However, the female patients whose reason of referral was the presence of disabling audio-vestibular symptoms had a peak in the peri-menopausal 5th and 6th decades. This group with disabling symptoms had a higher incidence in males [28]. They compared 80 patients referred for vestibulocochlear symptoms with 500 patients with multiple sclerosis for benign positional vertigo and Méniere’s [28]. Only migraineurs described cochlear sensations, such as tinnitus, distortion of pitch, or hearing loss [28].

The frequency of migraine in Méniere’s disease is higher than in normal subjects, and phonophobia has a high prevalence in these patients, independently of the presence of migraine headache [152].

### 4.1. Phonophobia

Phonophobia, along with photophobia, is one of the associated symptoms that define a migraine attack, according to the ICHD-3. As an asset for differential diagnosis, the presence of phonophobia may be able to exclude secondary headache types, such as cardiac cephalgia or sleep apnea headache; however, phonophobia is also reported in other headaches, such as a “tension-type headache”, if it is not accompanied by photophobia in the episodic categories, or a “cervicogenic headache”, which may make the clinician hesitate if the patient has a migrainous background [2]. This complication is simplified by using the appendix criteria for tension-type headaches that exclude both photophobia and phonophobia; and are clinically preferable.

In 1984, up to 81% of patients with migraine reported phonophobia, in comparison with only 12.1% of patients with a non-migrainous headache, and the combination of phonophobia and hearing loss was reported by some patients [28]. A recent meta-analysis showed that migraineurs may have a higher risk of developing sensorineural hearing loss [153]; therefore, the exclusion of migraine patients with hearing loss from the majority of the trials may lead to biased conclusions. In 1985, Blau and Solomon reported noise as a migraine trigger in 4/50 patients with migraine [133] and the potential measurability of phonophobia was suggested. Recently, it has been reported that annoying sounds, as well as other usually reported migraine triggers, may just represent early manifestations of migraine premonitory symptoms, as they demonstrate significant agreement with premonitory spontaneous phonophobia [154]. In studies that assessed sound discomfort using a range of Hertz stimuli, ictal [155,156] and interictal hearing discomfort thresholds were lower in migraineurs, as compared with healthy participants [156,157,158], with a low positive correlation with age [157]. Women may have a lower threshold than men [159]. Among migraineurs, ictal thresholds are lower than interictal ones [158]. Differences in monaural and binaural thresholds do not relate to the side of headache [156], and only a small proportion of participants with chronic migraine (5/48) report unilateral phonophobia, which was nonexistent in 54 participants with episodic migraine [160]. Similar to photophobia, unilaterality of phonophobia can be more specific to trigeminal autonomic cephalalgias [160].

The use of close-ended questions can be useful in increasing sensitivity for phonophobia during the neurological anamnesis [161].

Several electrophysiological studies have evaluated the hearing pathway in migraineurs with phonophobia. Phonophobia does not seem to be related with a recruitment phenomenon [155], which is commonly associated with cochlear damage.

The function of the cochlear efferents can be assessed by otoacoustic emission tests, which evaluates the suppression in the amplitude of transiently evoked signals from the olivary complex when a sound is produced on the contralateral ear [162,163]. It has been reported that for healthy controls, these amplitudes are significantly decreased, whereas in migraineurs, they are not suppressed [162,164]. This was specially observed in low-to-middle frequencies of 1–1.5 kHz, in a cohort of female phonophobic migraineurs during the interictal period [165]. However, this was not replicated in another study in patients with prominent vestibular symptoms, and phonophobia was not significantly associated with lack of suppression [163]. Neurotransmission in the outer hair cells of the cochlea may be mediated by CGRP [166], and increased CGRP activity in the inner ear has been hypothesized to be the cause of an insufficient suppression of the auditory pathway [165].

Another abnormality leading the patient to find sounds uncomfortable may lay in the cortical processing of auditory stimuli. Whereas latencies are similar, healthy participants experience a decrease in the amplitude of the auditory N1–P2 component following sequential blocks of stimuli in cortical-evoked auditory-evoked potentials, whereas participants with migraine experienced an increase, which could be considered a potentiation, instead of habituation. Intensity dependence of auditory-evoked potentials, which is measured as a slope after stimulation at increasing intensities, was also greater in migraineurs [159,167,168], and these may have a lower amplitude in the first blocks of stimuli, which may mean a decreased pre-activation of the sensory cortex [167,169]. The slope does not correlate with migraine frequency or duration, or with changes in visually evoked potentials [169], but may correlate with age [168], and has been associated with serotonergic activity [159,170,171,172] and response to preventive treatments [173].

Several studies have used brainstem auditory-evoked potentials. Interictal migraine patients have similar latency results to those of controls [174]. Podoshin et al. showed a significant impairment in interpeak latency differences in a group of patients during the migraine attack, when the rate of click sound stimuli was increased to 55 per second, in comparison with the same group between attacks [175]. Some studies found no differences between the side of the headache [175], but differences between sides were found by Schlake et al. in peak latencies at 10 clicks per second [174]. Peak latencies were delayed in 6/38 migraine patients, 2 of them with so-called basilar migraine [174], which can be normal [176] or abnormal during the ictal period [177]. Sand and Vingen showed that the discomfort threshold for low sound inversely correlated with low levels of habituation in wave IV-V, which corresponds with the lateral lemniscus in the pons and inferior colliculus in the midbrain [178]. Latency in waves III to V, corresponding to the tract between the cochlear nuclei to colliculus, has been correlated with migraine and attack duration [171]. In a recent study, participants with migraines showed that hearing threshold was inversely correlated with the severity of photophobia, and paradoxically, not with phonophobia, and was higher in patients on prophylactic medication or those who had taken a non-steroidal anti-inflammatory drug on the day of the test, and had higher wave amplitude in comparison with the controls [179].

There is increased blood flow in the auditory association cortex during an acute attack in patients with migraine and phonophobia [180].

Patients with episodic migraine that present with cranial and extracranial cutaneous allodynia have lower thresholds for auditory stimuli either between or during the attacks [181].

### 4.2. Vertigo

Vertigo is more frequent in people with migraine and vice versa [28,182,183,184,185,186,187].

Vestibular migraine (VM) is possibly the most frequent cause of recurrent vertigo [188]. It has received many names in the past [186,189,190], and recently, more conditions have been found to fall possibly under the current umbrella of what is considered today VM [191], as well as some diagnoses classified as functional disorders today that may, in the near future, be included. The mere fact of having a diagnosis has proven to be a positive predictor for the improvement of dizziness [192]. However, currently, VM still remains largely underdiagnosed [193]. Despite the consensus diagnostic criteria involving balance and headache societies [2,194], there are several mechanistic questions that remain unanswered, such as the controversy of whether migraine and VM are a continuum along the same spectrum or different entities, as well as important classification queries, such as whether there is a chronic form [195]. The current term of VM may not be well received by the patient, especially those examined outside a headache clinic environment, who usually do not report headaches as the main reason for referral [196], and a source of frustration for the clinician giving a diagnosis to patients who repeatedly report that they do not suffer from headaches.

The features of the attack of VM have been studied mainly retrospectively [186,189,190,197,198,199,200], and during the acute episode [201]. There may be a relationship between VM and Méniere disease (MD) [202]. Aural fullness may be an anamnestic key to differentiate VM from MD [203]. Patients with VM may have a high incidence of endolymphatic hydrops, although smaller than that of MD [204]; however, no anatomical differences were found between VM patients and healthy subjects with 3D-SPACE MRI [205]. A correlation between dizziness severity and cognitive dysfunction has been found [206].

Migraine and vestibular migraine: Similarities between migraine and VM are abundant. The majority (72/118) of patients with vestibular symptoms were considered in the 1980s as patients with “non-classical” migraine. Among those without vestibular symptoms, 59 out of 82 were given a diagnosis of “classical migraine”. The incidence of "classical migraine" was therefore 11% higher among those without vestibular symptoms [28]. Vertigo can be triggered with nitroglycerin in up to 84% of migraineurs reporting vertigo during spontaneous attacks [207]. Patients with migraines exhibit greater visual and vestibular functional impairment, as well as lower results in the sensory organization test [208]. VM patients may be more sensitive to moving scenes and find it harder to maintain their posture [209,210,211,212], as they may tend to rely more on visual cues [213], whereas changes in the position of the head or posture could also trigger vestibular symptoms in some migrainous patients [28].

Patients with definite vestibular migraines demonstrated some changes in videonystagmography, but not canal paresis [214]. Spontaneous nystagmus can be triggered in migraineurs following supraorbital nociceptive inputs, which did not occur following extracephalic stimulation of the median nerve [215].

Pathophysiology: The pathophysiological research that has used neuroimaging approaches has contributed enormously to understanding the central anatomical structures with altered function in VM. In a small study using ^18^F-deoxyglucose position-emission tomography, patients showed activation of the cerebellum, frontal cortices, thalami, dorsal pons and midbrain, right and insula and temporal cortex, and a deactivation of the posterior parietal and occipito-temporal areas during the attacks [216]. By using imaging-based voxel-based morphometry, patients with definite vestibular migraine showed a reduction in grey matter volume in several cortical areas, including the insula, parieto-occipital, dorsolateral prefrontal, cingulate cortex and the cingulate gyrus, and the volume of areas associated with vestibular and pain processing was negatively correlated with disease duration [217]. During caloric tests, patients with vestibular migraine exhibited increased thalamic activation, as observed in blood oxygenation level-dependent (BOLD) MRIs, which correlated with the attack frequency [218] and was proposed to hold right dominance [219]. A peripheral, vestibular alteration that involves serotonergic axons has also been suggested [220,221,222,223].

Treatment: Patient’s treatment remains a grey zone, where the therapeutic choice is dependent on observational studies, as there are only a few randomized, placebo-controlled trials in this field for preventive [224,225] and acute medication [29,226]. A recent meta-analysis identified an improvement in the outcomes selected for several therapeutic agents, most of them migraine preventives, such as tricyclics and beta-blockers [224]. Vestibular rehabilitation can also be of help [227].

Recently, the inhibition of CGRP receptors has been shown to improve the vestibular function in animal models of chronic migraines [228], and retrospective studies in humans show a potential benefit when targeting the CGRP pathway [229]. Half of the patients were reported to respond to one prophylactic, 17% responded to a combination of two, and 10% did not have a response [203]. Predictors of poor response have been reported to be female sex, interictal imbalance, anxiety or depression, and our next topic, cutaneous allodynia [203].

### 4.3. Allodynia

Scalp tenderness was reported by 65% of the 500 patients characterized by Selby and Lance in 1960, and they described that this sensitivity could not be correlated with any trigeminal or cervical radicular innervation [230]. Cutaneous allodynia can be quantified in humans objectively [231,232] or by assessing the subjective patient’s experience, by questionnaires [233,234]. A similar prevalence to that reported by Selby and Lance was found in large surveys of headache patients, slightly higher in those with the now obsolete term “transformed migraine”, and was associated with female sex, high body mass index or depression [235]. Up to one-fifth of patients report severe allodynic symptoms [234]. When specifically measured, the prevalence increases up to 80% [236] and can be higher in patients with another concomitant pain syndrome, such as temporomandibular disorders [237]. Patients with chronification of attacks and migraine with aura may also have a higher prevalence of cutaneous allodynia during the attack [238], although other studies have not found an association with age or headache frequency of years having migraine in migraineurs reporting spontaneous attacks [231,239].

*Pathophysiology*: The mechanisms that predispose a patient to allodynia may represent a risk for other forms of sensory dysfunction [164,203]. Migraineurs have, in general, lower pressure-pain and heat thresholds than the general population [232]. The majority of cutaneous allodynia symptoms are focused on the cranial regions, but a proportion can also experience the symptoms in extracranial regions [231]. In contrast to patients with migraine, patients with trigeminal autonomic cephalalgias, such as cluster headaches, do not report cutaneous allodynia, unless they have a personal or family history of migraine, and have higher pain threshold both interictally and during the attack [240]. Allodynia can be triggered experimentally in humans [239], and the clinical sequence of onset and anatomical spread has been described [241].

Allodynia was initially reported to be an ictal marker of a “no-return point” that divides triptan efficacy [242]; however, triptans can treat spontaneous [243] and nitroglycerine-induced allodynia associated with migraine in humans [239], and the association appeared to be, instead, time-dependent [244,245]. In a similar way to low pain intensity, which can also increase as the attack progresses, lower allodynia may be an independent predictor for the efficacy of over-the-counter acute treatments [246], and recently, allodynia has been shown to be an independent risk factor for the worsening of migraine associated with the utilization of masks during the COVID-19 pandemic [247].

A complex network of peripheral and central structures is involved in allodynia. In 1994, reduced efficacy in the spinal inhibitory circuits, mediated by GABA-A, was proposed as a potential cause of allodynia in preclinical models of pain [248]. Two years later, it was shown that trigeminal afferents could be sensitized with a variety of chemical substances applied in the dural regions [249]. However, it is unlikely that the simple sensitization of peripheral afferents accounts for the single cause of allodynia. The periaqueductal grey holds inhibitory control over trigeminal afferent neurons [250,251] and also has a regulatory effect on the trigeminocervical nucleus (Figure 1), facilitated by CGRP [252]. Another neuropeptide, pituitary adenylate cyclase-activating peptide 38 (PACAP-38) can cause sensitization and delayed activation of trigemino-cervical neurons [253]. Under the bases of the role of the trigemino-cervical complex as a convergence center for afferent inputs [254], and its diencephalic connections, an increased response in central neurons could bring a reduced pain threshold in extracranial regions [255].

The diencephalon may be, indeed, strongly involved in the process of allodynia. Stress-related hypothalamic dysregulation of prolactin has recently been associated with allodynia in females [256]. Activation in posterior thalamic areas was demonstrated in rodents and also in migraine patients with extracephalic allodynia, with functional MRI BOLD techniques [257]. A first-line treatment in the prevention of migraine, propranolol, exerts part of its mechanisms upon these thalamic areas [258]. Thalamic projections are widely spread to many areas of the cortex, and have been traced from posterior and lateral nuclei to several cortical regions, including the auditory, entorhinal or visual cortex [259]. The medial area of the temporal lobe, for example, may be hyperexcitable in migraineurs, both during ictal and interictal moments, when applying painful heat stimuli to the forehead, as detected with diffusion tensor imaging in functional MRI [260]. An hyperexcitable state has also been suggested in subcortical regions in migraineurs [261].

Somatosensory-evoked potentials have not found significant abnormalities in migraineurs [262]. However, cortical thickness may be different in the associated temporo-parietal areas of migraineurs, and there is a positive correlation with pain threshold, contrary to healthy controls [263,264]. Activity is also increased in primary sensory areas, and between the pons and insula, implying a role in the patient’s emotional response [265].

In preclinical models of allodynia, nitroglycerine is capable of increasing the firing of trigeminal neurons and dural-evoked action potentials, in addition to creating hypersensitive responses to facial stimulation with innocuous brush or noxious pinch. These responses were reversible with naratriptan [239] and also ibuprofen, suggesting both a serotonin and an inflammatory-mediated mechanism [266,267].

Allodynia has not been directly related to levels of amylin or CGRP [268]; however, it can be modulated to target CGRP [269,270,271], which may have a glial site of action [272] and stronger activity in females [271]. Nitroglycerine was able to trigger allodynia in 17/53 patients with migraine; among them, 14 responded to acute treatment with aspirin or sumatriptan, and those who reported allodynia in their usual attacks were more likely to experience it during the triggering session [239].

Finally, TRP channels are an interesting area in the understanding and treatment of migraine [273]. Migraineurs have less tolerance to heat during the interictal period [274]. Recent studies did not find an association between thermal quantitative sensory testing (QST) and allodynia. However, preclinical models suggest a potential genetic predisposition to mechanical allodynia, involving the non-selective cold-sensitive cation channel transient receptor potential melastatin 8 (TRPM8), the activation of which causes cranial and extracranial allodynia [275]. Fibres that express TRPM8 were progressively reduced in postnatal mice, in contrary to the fibres that express CGRP. Paradoxically, the use of the TRPM8 agonist menthol can reduce behavioural responses to meningeal chemical stimulation [276]. These channels may have potential hypothalamic modulation, as orexins may play a part in the emotional response to heat [277]. It may be speculated that these differences could potentially translate to different phenotypes of migraineurs, which find relief either with fresh air or a heated pad.

## 5. Conclusions

This article summarizes the literature regarding the associated symptoms in migraineurs. Knowledge concerning migraines and their associated symptoms continues to grow and is evolving into a concept that might not be as clinically simple as once imagined [278], with a wide spectrum of presentations of the same migrainous biology. Trials that have reported the most bothersome associated symptoms, together with pain, represent a more holistic approach to migraine research.

Associated symptoms of migraine are varied, extremely prevalent, and contribute to the disabling nature of migraines. Acknowledging the associated symptoms could contribute to a better outcome for the patient, and should never be forgotten in the anamnesis of the migraineur. Treatment should be focused on correct acute, preventive and anti-emetic migraine treatments, where needed.

The relationship between the central and peripheral sensitization processes with the associated symptoms of migraines is evident, and is comparable to the question of what was first to come, the chicken or the egg.

## Data Availability

Not applicable.
